# The treatment of malignant glaucoma in nanophthalmos: a case report

**DOI:** 10.1186/s12886-018-0714-5

**Published:** 2018-02-23

**Authors:** Jie Wang, Ergang Du, Jinfei Tang

**Affiliations:** 1 Department of Ophthalmology, Zhejiang Chinese Medical Hospital, Hangzhou, 31006 China; 2 Department of Ophthalmology, Zhejiang Provincial Traditional Chinese Medical Hospital, 54 Youdian Road, Hangzhou, 310006 China

**Keywords:** Malignant glaucoma, Cataract, Nanophthalmos

## Abstract

**Background:**

The management of eyes with nanophthalmos is a dilemma for ophthalmologists due to various complications, especial the eye with malignant glaucoma. We report a case of effective treatment for malignant glaucoma in nanophthalmos.

**Case presentation:**

An 82-year-old man was performed phacoemulsification in the right eye with normal ocular pressure and nanophthalmos. The surgery was uneventful: an intraocular lens (IOL) was placed and centered in the capsular bag. 2 months later, the patient presented with malignant glaucoma, and the intraocular pressure fluctuated between 18.6 mmHg and 30.8 mmHg with antiglaucoma medications. The patient did not respond to surgical peripheral iridotomy and goniosynechialysis. Then a single treatment with laser peripheral lens posterior capsulotomy and vitreous anterior membranectomy was performed. The intraocular pressure normalized, and the anterior chamber deepened within 24 h. The patient’s condition remained stable for 9 months with no further treatment, and his Snellen corrected distance visual acuity was 20/50. The left eye of this patient was treated by combined surgery including phacoemulsification, IOL implantation, anterior vitrectomy, surgical peripheral iridotomy (PI), and goniosynechialysis. No intraoperative or postoperative complications were observed.

**Conclusions:**

This case suggests that it is essential to choose a suitable treatment for nanophthalmos patients to deal with malignant glaucoma and to reduce the incidence of malignant glaucoma.

## Background

Nanophthalmos is part of the clinical spectrum of microphthalmos, which is typically a small eye without ocular malformations. Nanophthalmos is characterized by a short axial length [1], thickened sclera [2], a shallow anterior chamber, and a normal-to-large lens with a high lens-to-eye-volume ratio [3]. These basic clinical features account for glaucoma complication in these eyes. Specifically, the lens is of normal volume but is disproportionately large than the sized of the small eye [4].

Malignant glaucoma is described as normal or elevated intraocular pressure (IOP) associated with axial shallowing of the entire anterior chamber in the presence of a patent peripheral iridotomy (PI). It is also known as ciliary block glaucoma or aqueous misdirection glaucoma. It typically developed after glaucoma drainage surgery in patients with a history of angle-closure glaucoma (ACG). However, it can also occur after phacoemulsification [5], and initiation caused by topical miotic therapy [6], or in eyes that have not previously undergone surgery [7]. We describe our successful use of laser peripheral capsulotomy in a patient with a nanophthalmic eye who developed malignant glaucoma after phacoemulsification. The second eye with cataract of this patient was treated with phacoemulsification combined with anterior vitrectomy, surgical PI, and goniosynechialysis.

## Case presentation

An 82-year-old man underwent uncomplicated phacoemulsification with implantation of a posterior chamber intraocular lens in his right eye. The continuous curvilinear capsulorhexis (CCC) was successful performed, and the IOL was centered in the capsular bag. The IOP was normal, and his Snellen best corrected visual acuity (BCVA) was 20/40 at the first day postoperatively. 20 years earlier, he had undergone bilateral neodymium: YAG (Nd: YAG) PI to treat narrow drainage angles and had persistently shallow anterior chambers with normal IOP after the Nd: YAG procedure. After 2 months postoperative, the IOP in the right eye increased. The patient received two antiglaucoma medications (i.e., Travatan once a day and 2% carteolol twice a day) to control the IOP, but it remained high. The IOP fluctuations ranged from 20.5 to 27.6 mmHg. He was then referred to our hospital.

**Fig. 1:**
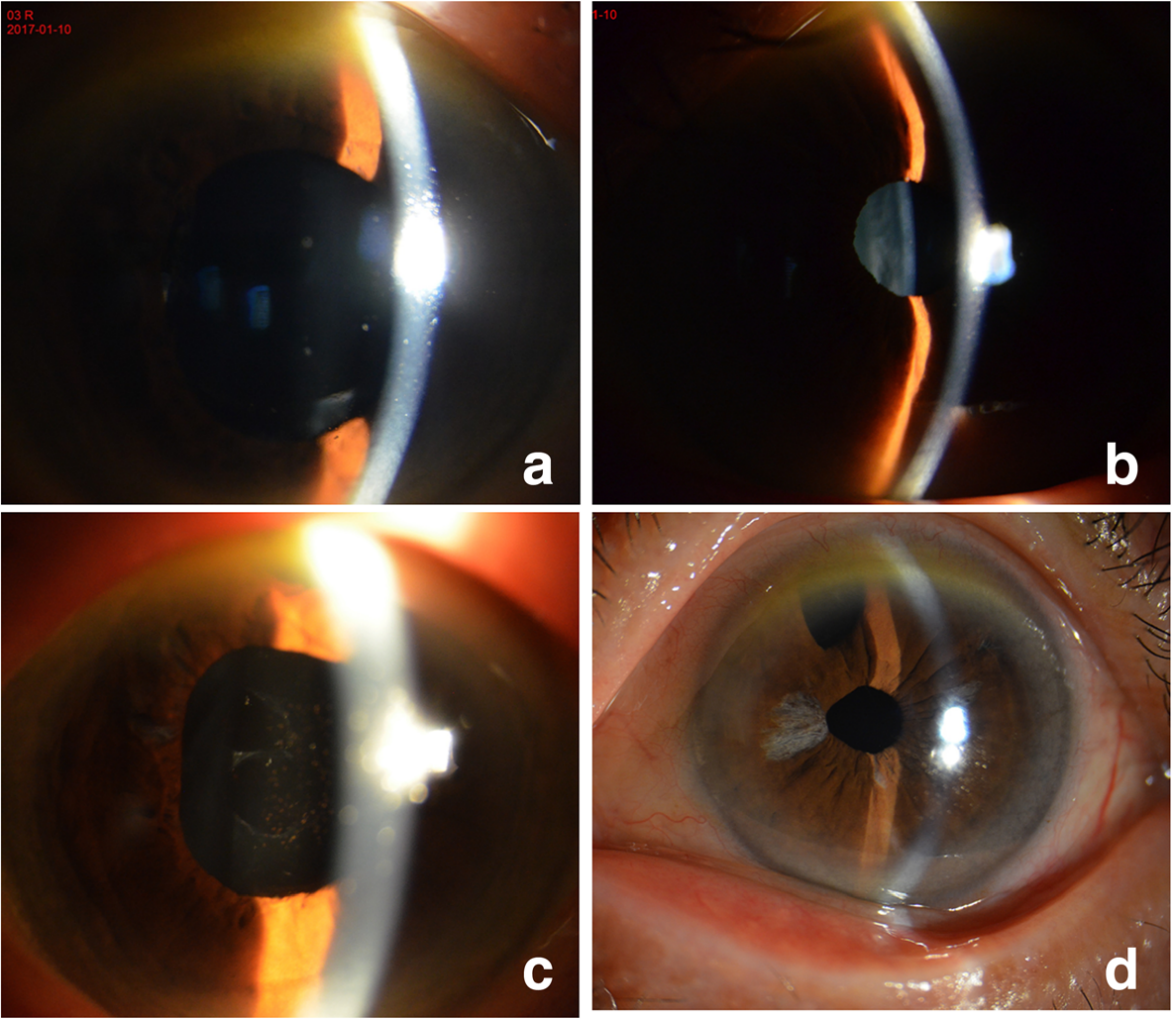
Shallow anterior chamber observed by slitlamp biomicroscopy in the right eye (**a**) and in the left eye (**b**). Deep anterior chamber observed after laser treatment in the right eye (**c**) and surgery in the left eye (**d**)

**Fig. 2:**
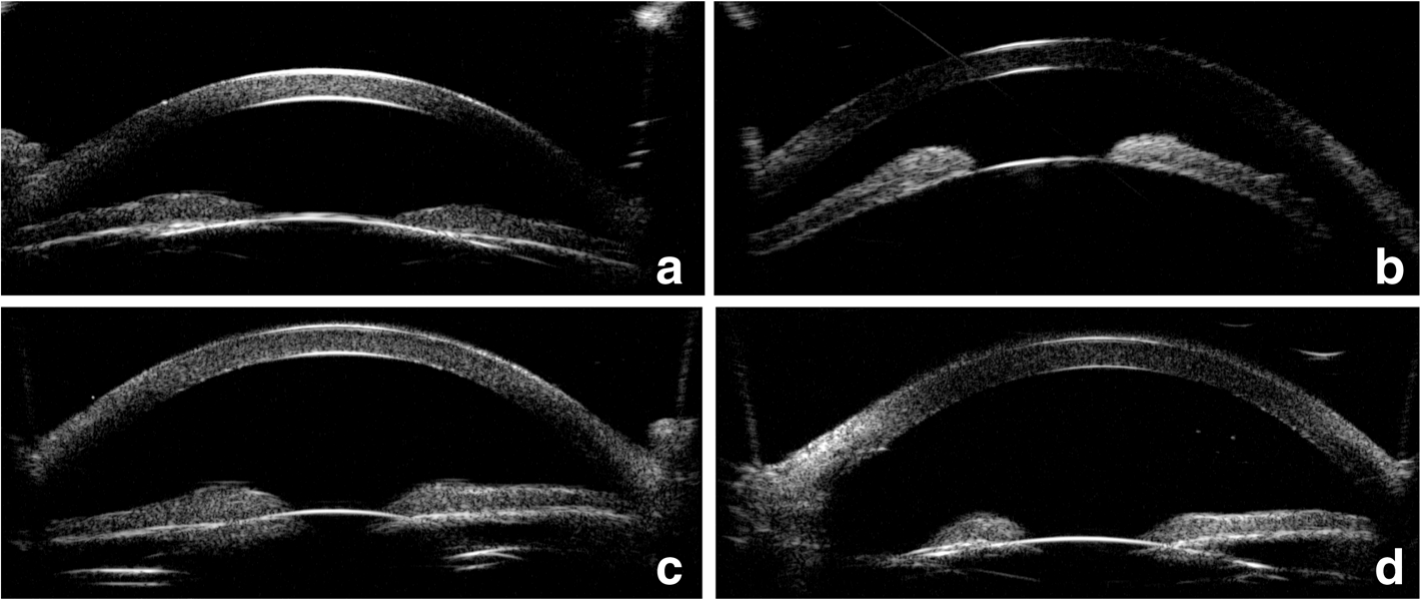
Shallow anterior chamber observed by UBM in the right eye (**a**) and in the left eye (**b**). Deep anterior chamber observed after laser treatment in the right eye (**c**) and surgery in the left eye (**d**)

As malignant glaucoma was highly suspected in the right eye, ultrasound biomicroscopy (UBM), axial scan, slit lamp, and pharmacologic mydriasis were used to confirm the diagnosis. The patient’s both eyes were small, with an anterior chamber depth/axial length of 2.14/20.51 mm in the right eye, and 1.61/20.70 mm in the left eye. Slit lamp revealed a mild conjunctival hyperemia and corneal edema, a patent PI was present in the temple superior, and the anterior chamber was shallow (Fig. 1, A, B). Ultrasound biomicroscopy showed extremely shallow anterior chamber (Fig. 2, A, B). Pharmacologic mydriasis was performed. The IOP decreased from 27.5 to 22 mmHg after 30 min of application of compound tropicamide eyedrop. However, the IOP increased to 30.8 mmHg again after 6 h of application of tropicamide. A diagnosis of malignant glaucoma in nanophthalmos was made.

Peripheral iridotomy and goniosynechialysis were performed by the surgeon. Goniosynechialysis was performed using a 1 ml syringe needle tip under the observation of a gonioscope. Then, we used capsule scissors to cut off 1 mm × 1 mm sized iris tissue to build a channel between the anterior and posterior chamber at the preexisting peripheral iris defect. The first day after surgery, the IOP in the right eye was 20.0 mmHg and the anterior chamber remained shallow. Thus, we preformed YAG laser lens posterior capsulotomy in the central pupil zone, 4 h after YAG laser, the IOP was 19.7 mmHg. Then the IOP in the right eye increased uncontrollably from 22 to 33.1 mmHg. He received two kinds of anti-glaucoma medications (i.e., Travatan once a day and 2% carteolol twice a day) to control the IOP. However, it remained high. 5 days after PI and goniosynechialysis, the right eye underwent YAG laser lens posterior capsulotomy and vitreous anterior membranectomy in the temple superior where surgical PI had been performed. The IOP decreased from 31.1 mmHg to 21.1 mmHg within 6 h, and the anterior chamber became deep (Fig. 1C, Fig. 2C). At the last follow-up visit, 9 months after the YAG laser lens posterior capsulotomy and vitreous anterior membranectomy, the BCVA was 20/50 with + 1.00–1.25 × 106, the IOP was controlled at 11.2 mmHg, and the anterior chamber depth was 2.87 mm.

As the right eye appeared to have a series of complications after simple phacoemulsification and IOL implantation, we preformed combined surgery in the left eye. A clear corneal temporal incision was made. After a continuous curvilinear capsulorrhexis approximately 5.5 mm in diameter was created, phacoemulsification was performed. Then, goniosynechialysis was performed using a 1 ml syringe needle tip under the observation of a gonioscope. After performed goniosynechialysis, an infusion cannula was placed in the vitreous cavity through pars plana. Then posterior capsulorhexis was performed with the tip of a standard 23-gauge bent cystotome and the anterior vitreous was also excised. The anterior chamber deepened visibly during vitrectomy. We also preformed an iridectomy with 23-gauge bent cystotome at the superior iris, then, posterior lens capsule, part lens zonule, and vitreous anterior membrane were cut in the superior to create an open pathway between the anterior chamber and the vitreous cavity. We determined that anterior chamber could be rapidly deepened after discharging the aqueous humor of the anterior chamber, and that the channel was open. Then a foldable single-piece posterior chamber IOL (Akreos MI60, Bausch) was inserted into the capsular bag. 7 months after surgery, the BCVA was 20/50 with + 1.50–1.0 × 91, the IOP was controlled at 12.3 mmHg, and the anterior chamber depth was 2.74 mm. (Fig. 1D, Fig. 2D).

## Discussion

The complications of nanophthalmos include malignant glaucoma, uveal effusion syndrome with or without exudative retinal detachment, and acute ACG [8, 9]. Several theories can explain the occurrence of malignant glaucoma in nanophthalmos. Aqueous misdirection is the anterior displacement of the lens-iris and iris-hyaloid diaphragm [10]. The ciliolenticular theory is characterized by the anterior rotation of the ciliary body [5]. Any intervention for malignant glaucoma aims at directly communicating between the anterior chamber and the vitreous cavity to disrupting the blockage site at the ciliary-hyaloid interface. Nd: YAG laser therapies and vitreous surgical interventions are well reported in the literature [5]; but inappropriate use of these treatments may result in ineffective.

In our case, after we diagnosed nanophthalmos with malignant glaucoma, we performed surgical PI to relieve the papillary block and goniosynechialysis to open the closed angles. Unfortunately, the management of elevated IOP was ineffective with PI and goniosynechialysis. Then, we preformed YAG laser lens posterior capsulotomy in the central pupil zone. However, it’s still ineffective. We had to perform lens posterior capsulotomy and vitreous anterior membranectomy simultaneous with Nd:YAG laser in the temple superior where surgical PI had been performed, and the IOP decreased. We speculated that YAG laser disrupted the mechanism of aqueous misdirection, the misdirection of aqueous fluid flowed from the posterior segment to anterior segment through the temple superior passageway. The aqueous humor secreted by the ciliary bady is difficult to reach the papillary region through the thick vitreous, and then flow into anterior chamber. So YAG laser lens posterior capsulotomy in the central pupil zone may be ineffective. But Nd:YAG laser peripheral capsulotomy allows aqueous humor to flow into anterior chamber easily. Papillary block was effectively relieved. Although the IOP was eventually controlled, patients underwent multiple surgeries that led to more pain and expenses. Therefore, phacoemulsification was considered necessary to deal with the cataract, but additional laser or surgical maneuvers were required to avoid intraoperative and postoperative complications of nanophthalmos.

There is no gold standard for the treatment of malignant glaucoma in nanophthalmos patients. Combining vitrectomy with phacoemulsification has been suggested as a strategy to reduce the intraoperative risks in eyes with a shallow anterior chamber [11]. The controlled removal of the anterior vitreous facilitates the posterior displacement of the lens, deepens the anterior chamber, and decreases the IOP [12]. The removal of the anterior vitreous can decrease the positive vitreous pressure. At the same time, the anterior vitrectomy can open the iris-hyaloid diaphragm and relieve the ciliary block. On the basis of these outcomes, we performed phacoemulsification combined with anterior vitrectomy, surgical peripheral iridotomy and goniosynechialysis when we diagnosed his left eye was nanophthalmos and cataract. We cut posterior lens capsule, part lens zonule, and vitreous anterior membrane in the superior to create an open pathway between the anterior chamber and the vitreous cavity, and to avoid ciliary block. We determined that anterior chamber could be rapidly deepened after discharging the aqueous humor of the anterior chamber, and that the channel was open. That is the issue. We make sure that the channel is open, and the operation is successful. The outcome of our case suggests that phacoemulsification cataract surgery combined with anterior vitrectomy, surgical PI, and goniosynechialysis can achieve a positive prognosis in nanophthalmic patients with cataract and suspicious malignant glaucoma. Malignant glaucoma after the phacoemulsification cataract surgery of nanophthalmic eyes can be treated by laser peripheral capsulotomy and vitreous anterior membranectomy. Combined surgery is safe and effective.

## Conclusion

Our case showed that malignant glaucoma is a common complication of nanophthalmos patients. It is important to choose an appropriate treatment for nanophthalmos patients to deal with malignant glaucoma and to reduce the incidence of malignant glaucoma.

## Abbreviations

ACG: Angle-closure glaucoma
BCVA: Best corrected visual acuity
CCC: Continuous curvilinear capsulorhexis
IOL: Intraocular lens
Nd: YAG: Neodymium: YAG
PI: Peripheral iridotomy
UBM: Ultrasound biomicroscopy
